# Changes of Dendritic Spine Density and Morphology in the Superficial Layers of the Medial Entorhinal Cortex Induced by Extremely Low-Frequency Magnetic Field Exposure

**DOI:** 10.1371/journal.pone.0083561

**Published:** 2013-12-20

**Authors:** Jiaxiang Xiong, Chao He, Chao Li, Gang Tan, Jingcheng Li, Zhengping Yu, Zhian Hu, Fang Chen

**Affiliations:** 1 Department of Physiology, Third Military Medical University, Chongqing, PR China; 2 Department of Occupational Health, Third Military Medical University, Chongqing, PR China; Peking University, China

## Abstract

In the present study, we investigated the effects of chronic exposure (14 and 28 days) to a 0.5 mT 50 Hz extremely low-frequency magnetic field (ELM) on the dendritic spine density and shape in the superficial layers of the medial entorhinal cortex (MEC). We performed Golgi staining to reveal the dendritic spines of the principal neurons in rats. The results showed that ELM exposure induced a decrease in the spine density in the dendrites of stellate neurons and the basal dendrites of pyramidal neurons at both 14 days and 28 days, which was largely due to the loss of the thin and branched spines. The alteration in the density of mushroom and stubby spines post ELM exposure was cell-type specific. For the stellate neurons, ELM exposure slightly increased the density of stubby spines at 28 days, while it did not affect the density of mushroom spines at the same time. In the basal dendrites of pyramidal neurons, we observed a significant decrease in the mushroom spine density only at the later time point post ELM exposure, while the stubby spine density was reduced at 14 days and partially restored at 28 days post ELM exposure. ELM exposure-induced reduction in the spine density in the apical dendrites of pyramidal neurons was only observed at 28 days, reflecting the distinct vulnerability of spines in the apical and basal dendrites. Considering the changes in spine number and shape are involved in synaptic plasticity and the MEC is a part of neural network that is closely related to learning and memory, these findings may be helpful for explaining the ELM exposure-induced impairment in cognitive functions.

## Introduction

The extremely low-frequency magnetic field (ELM) is produced by power lines used in transmission and distribution of electric power and by the numerous appliances (TV sets, PC monitors, mobile phones, etc.) used in houses and work places [1]. As the widespread use of electricity in modern society, it is impossible to avoid exposure to ELM in our environment. There is increasing evidence that exposure to ELM can influence learning and memory, although some negative results have been reported [2], [3]. Previous studies have shown that ELM exposure impaired acquisition and consolidation of spatial memory in rodents [4]–[6] and produced deficits in detour learning and one-trial passive avoidance learning in chicks [7], [8]. Moreover, it has been reported that exposure to ELM impaired the performance of humans in word recognition and visual discrimination tasks [9], [10].

The entorhinal cortex (EC) is closely related to learning and memory and has long been viewed as the interface between cortical regions and hippocampus [11]. The superficial layers (I–III) of the EC are regarded as ‘input layers’ since they receive convergent sensory inputs from perirhinal and parahippocampal cortices and then project to all the subregions of the hippocampus [12], [13]. The EC has been subdivided into two subregions, the medial entorhinal cortex (MEC) and the lateral entorhinal cortex (LEC). The MEC contains the functional neurons with spatial firing properties, including grid cells, head direction cells, grid × head direction conjunctive cells, and border cells and is a part of neuronal circuits that aid in the spatial learning and memory [14]–[16]. Furthermore, dysfunction of the EC is associated with the development of Alzheimer's disease and schizophrenia, both of which are characterized by impairments in learning and memory [17], [18].

Dendritic spines are the bulbous protrusions that form the postsynaptic part of excitatory synapses in the central nervous system (CNS) and their structure and density are crucial determinants of neuronal input-output transformations [19]. In CNS, they provide an anatomical substrate for memory storage and synaptic transmission. The gain, loss, and morphological remodeling of dendritic spines are implicated in learning and memory [20], [21]. The principal neurons in the superficial layers of the MEC contain tens of thousands of spines that receive mostly excitatory inputs from other neurons, however, little is known about the potential effects of exposure to ELM on dendritic spines in this brain region in rats.

In the present study, we have determined the temporal effects of ELM exposure on spine density and shape in the dendrites of stellate neurons as well as pyramidal neurons in the superficial layers of the MEC by performing Golgi staining. Considering that MEC is closely related to spatial learning and memory, thus to determine the changes in dendritic spines in MEC might be helpful for providing a neural mechanism for the effects of ELM exposure on cognition.

## Materials and Methods

### Subjects and Their Treatment

A total of 20 male Sprague-Dawley rats (about 150 g at the beginning of the experiment, n =  5/group) were used for this study. The animals were housed in groups of five per cage with natural light cycle (12-h light: 12-h dark cycle, lights on at 7:00 A.M.) and constant room temperature (24±2°C). Water and food were available ad libitum. All procedures were conducted in accordance with the National Institutes of Health Guide for care and use of laboratory animals and were approved by the Third Military Medical University Animal Care Committee. The rats were randomly assigned into four treatment groups: (1) no ELM exposure (control 14 days group); (2) exposure to ELM 4 h/day for 14 days; (3) no ELM exposure (control 28 days group); (4) exposure to ELM 4 h/day for 28 days.

### Magnetic Fields Exposure System

The exposure system used in our study was similar to the one used in other studies [22]. In brief, ELM was generated by using a round coil electromagnet with a 1000 turned copper wire. The electromagnet was supplied with sinusoidal current (50 Hz), the intensity of which was controlled by a power regulator. The magnetic induction of magnetic field was measured with a Gauss meters. When adjusted the current intensity, magnetic field with average magnetic induction of 0.5 mT could be generated in the center of the coils.

During ELM exposure, four groups of rats were simultaneously placed in their usual plastic cages. The centre of each cage was 15 cm from the poles and exposed with an average magnetic induction of 0.5 mT. The ELM exposure was conducted repeatedly every afternoon (2:00–6:00 P.M.). The conditions for control groups were the same as for the exposed groups but without the magnetic field.

### Golgi Staining

Golgi staining was carried out using the FD Rapid Golgi Stain Kit (FD Neurotechnologies, Baltimore, MD) and the methods had been detailed previously [23], [24]. In Brief, the removed rat brains were placed in an appropriate impregnation solution and stored in the dark for 14 days at room temperature. The brains were then transferred into a sucrose-based solution and stored at 4°C for 2–7 days. Finally, semi-horizontal sections (150 µm) containing the EC were prepared with an oscillating tissue slicer (Leica, VT1000, Wetzlar, Germany) and mounted on gelatin coated slides. Slides were then allowed to air dry at room temperature. Once sufficiently dry, sections were rinsed in distilled water and placed in a solution containing silver nitrate for 10 min. Slides were then rinsed again in distilled water and dehydrated in absolute alcohol, cleared with xylene, mounted on slides and covered with non-acidic synthetic balsam and coverslips.

### Data Acquisition

All image acquisition and analyses were performed in a blind manner. Compared the slices to the atlas (Paxinos-Watson, 1998), we choose the slices of Bregma–4.0 – –8.0 to be observed. The images for spine analysis were obtained using a Leica microscope with a 1000 x magnification. To assure a homogenous neuronal population, the neurons that selected for analysis fulfilled the following criteria: (1) the cell body and dendrites were completely impregnated; (2) the neurons were isolated from the surrounding neurons; (3) all the dendrites were visible within the plane of focus. We reconstructed intact cell morphology, and calculated dendrite length by using imaging analysis software (cellSens Standard 1.7). Dendritic spines were counted on 20 µm of second-order dendrites of stellate neurons as well as apical and basal dendrites of pyramidal neurons in the superficial layers of MEC. The spine density was expressed as the average number of spines per micron of dendrite length.

### Statistical Analysis

All data were presented as Mean ± SEM. An unpaired two-tailed t-test was used to evaluate statistical differences between control and ELM exposure groups. Significant differences were accepted at *P*<0.05.

## Results

### ELM Exposure does not Affect the Total Dendrite Length of the Principal Neurons in the Superficial Layer of the MEC

**Figure 1:**
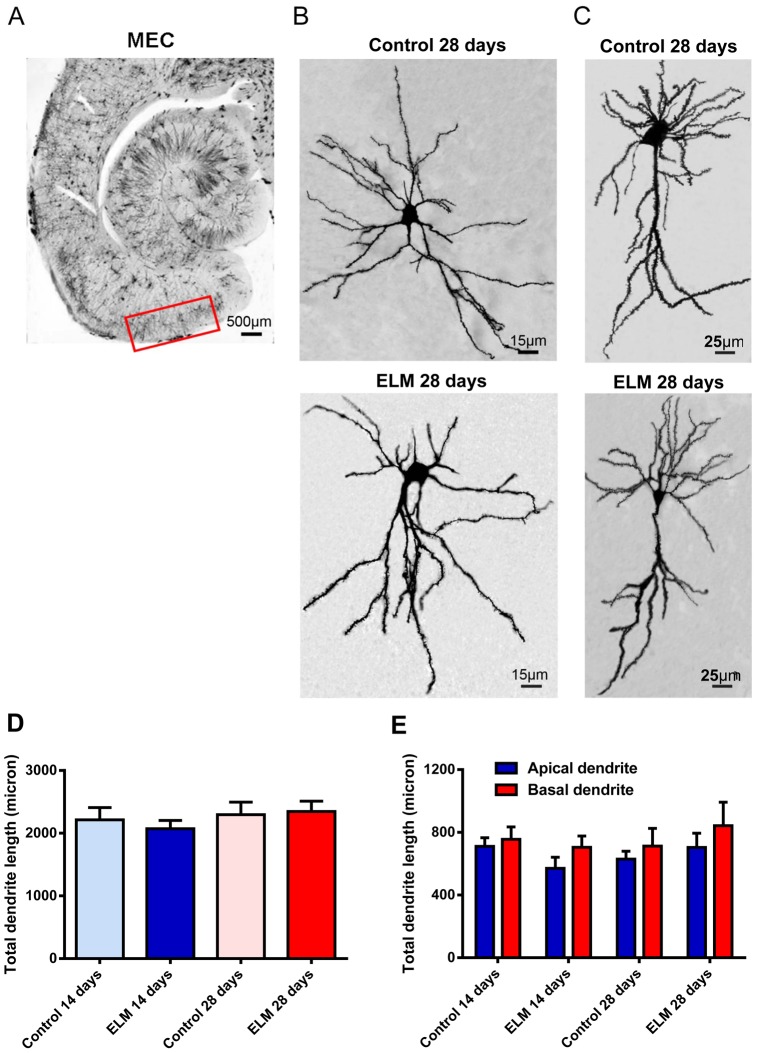
Extremely low-frequency magnetic field (ELM) exposure does not influence the total dendrite length of the principal neurons in the superficial layers of the medial entorhinal cortex (MEC). (A) The images taken with microscope show the place of superficial layers of rat MEC in brain section. (B-C) The morphology of Golgi-impregnated stellate (B) and pyramidal (C) neurons of control and 28-day ELM exposure. (D-E) ELM exposure did not alter the total length of dendrites in stellate neurons (D) as well as apical and basal dendrites in pyramidal neurons (E) in the superficial layer of the MEC.

Stellate and pyramidal neurons are the major types of excitatory projection neurons in the superficial layers of the EC [25], [26]. In the present study, we identified two types of neurons by their morphology and location. Stellate neurons in layer II or the border of layer II and III had oval somata with multiple thick primary dendrites that radiated out from the cell body, but lacked a clearly dominant dendrite (Fig. 1B). Pyramidal neurons in layer II/III had a pyramidal soma with one thick apical dendrite and several basal dendrites extended toward the deeper layers (Fig. 1C). The somata of pyramidal neurons were smaller than the stellate neurons.

Firstly, we investigated the effect of ELM exposure on total dendrite length of the principal neurons. There was no significant change in the total dendrite length of stellate neurons at both 14 days (*P* = 0.55, Fig. 1D) and 28 days (*P* = 0.85, Fig. 1B and 1D) after ELM exposure. Considering the apical and basal dendrites of pyramidal neurons have different connectivities and biophysical properties [27], total dendritic length and spine were analyzed separately in the apical and basal dendrites. Similar to stellate neurons, ELM did not affect the total length of apical and basal dendrites in pyramidal neurons at both 14 days (apical dendrite: *P* = 0.12; basal dendrite: *P* = 0.66; Fig. 1E) and 28 days (apical dendrite: *P* = 0.45; basal dendrite: *P* = 0.73; Fig. 1C and 1E) post exposure.

### ELM Exposure Reduces the Thin, Branched but not Stubby Spine Density of the Stellate Neurons

**Figure 2:**
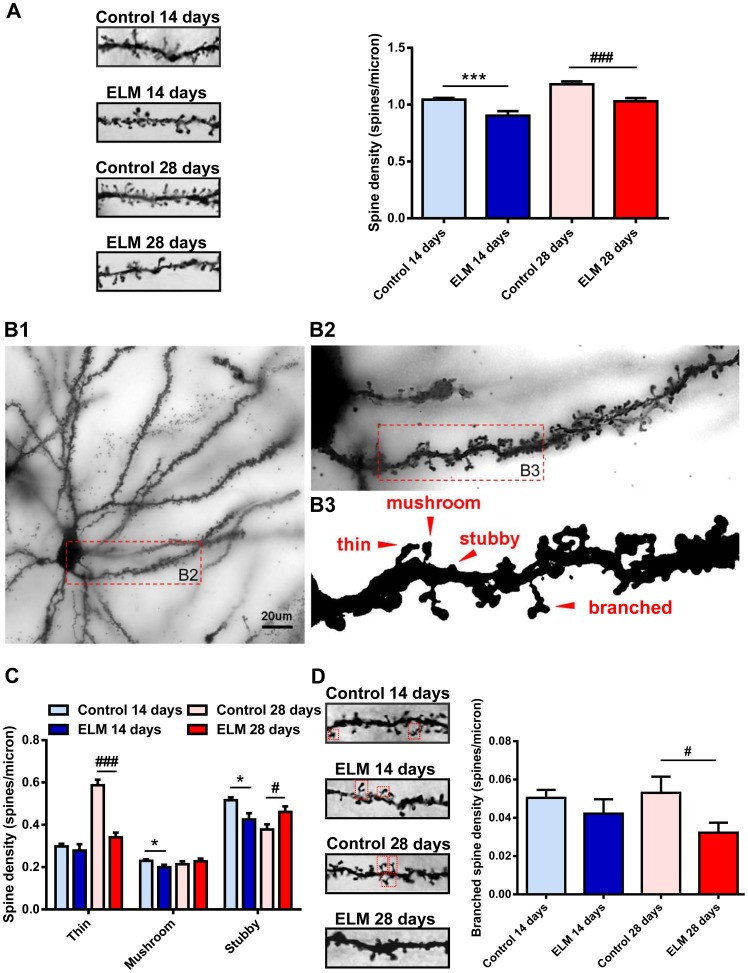
ELM exposure reduces the thin, stubby, branched but not mushroom spine density of the stellate neurons. (A) Representative images of spines on the 20 µm second-order dendrites of stellate neurons show that the spine density was reduced at 14 days and 28 days post ELM exposure (left). The histogram summarizes the effect of ELM exposure on spine density in stellate neurons (right). (B1-B3) Representative images of spine subtypes classified by morphology according to the ratio of spine head width to spine neck width and the number of spine head. (C) Bar histogram shows the group data of the effect of ELM exposure on the thin, mushroom and stubby spine density of stellate neurons. (D) Representative images of branched spines (shown in a red frame) on the 20 µm second-order dendrites of stellate neurons of control and ELM exposure group at 14 and 28 days respectively (left). Pooled data show that ELM decreased the branched spine density only at 28 days post exposure while the changes observed earlier were not statistically significant (right). *P<0.05; ***P<0.001; ^#^ *P*<0.05; ^###^ *P*<0.001.

For the stellate neurons, dendritic spine density was significantly reduced at both 14 days (86.42±2.77% of control, *P*<0.001, Fig. 2A) and 28 days (87.34±1.10% of control, *P*<0.001, Fig. 2A) after the ELM exposure. The variable spine shape is thought to be correlated with the spine stability and synaptic strength. Dendritic spines with large heads are highly stable and contribute to strong synaptic connections, while spines with small heads are generally transient and form weak synaptic connections [21], [28]. Next, we investigated the effect of ELM exposure on the spine shape. According to the ratio of spine head width to spine neck width and the number of spine head, spines were classified into one of the three morphological categories (Fig. 2B3): 1) Mushroom spine: big spines with a large head and mushroom appearance; 2) Thin spine: spines with a long neck and small head; and 3) Stubby spine: very short spines without a notable neck. There was a significant change in the density of stubby spines at 14 days (82.32±2.24% of control, *P*<0.05, Fig. 2C) and 28 days (121.96±1.07% of control, *P*<0.05, Fig. 2C) after ELM exposure. And there was a significant reduction in the density of thin spines at 28 days (58.04±0.85% of control, *P*<0.001, Fig. 2C) after ELM exposure in stellate neurons. We did not observe a significant change in the density of thin spines at 14 days (93.35±2.41% of control, *P* = 0.55, Fig. 2C) after ELM exposure in stellate neurons. Moreover, there was a significant reduction in the density of mushroom spines at 14 days (87.11±0.16% of control, *P*<0.05, Fig. 2C). While no significant change was observed in the density of mushroom spines at 28 days (106.54±0.91% of control, *P* = 0.45, Fig. 2C) after ELM exposure. Branched spines were a subtype of mushroom spines that bifurcate into two or more branches at variable points from the origin (Fig. 2B3). Considering the number of branched spines correlates with long-term potentiation (LTP) induction [29], we specially investigated the effect of ELM on this type of spines. Interestingly, the density of branched spines was decreased at 28 days (60.75±0.62% of control, *P*<0.05, Fig. 2D) post ELM exposure, while the changes observed earlier were not statistically different (83.73±1.80% of control, *P* = 0.36, Fig. 2D).

### ELM Exposure Primarily Alters the Dendritic Spine Density and Shape in Basal Dendrites of the Pyramidal Neurons

**Figure 3:**
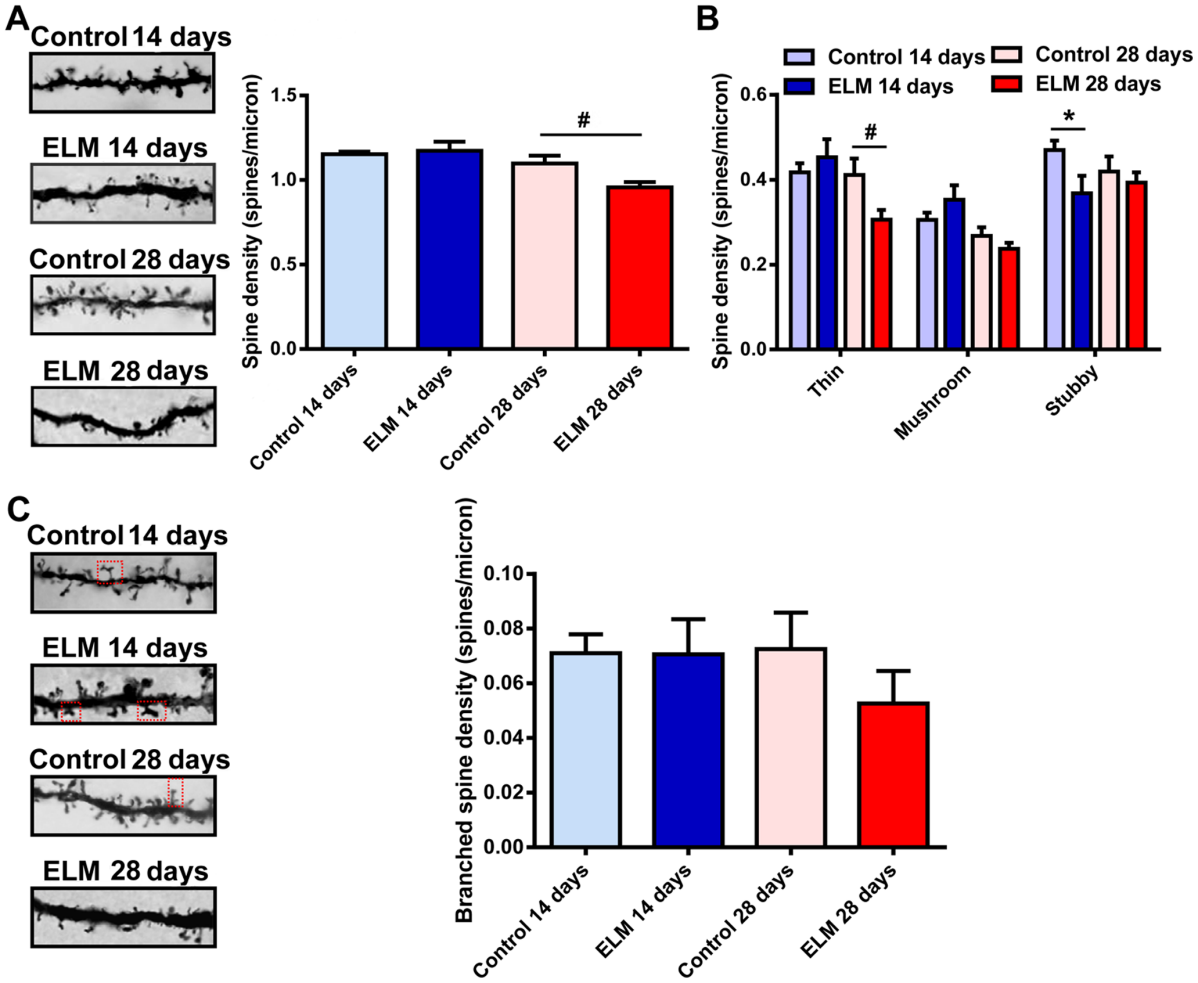
ELM exposure influences the spine density and shape in the apical dendrites of pyramidal neurons. (A) Representative images of spines on the 20 µm second-order apical dendrites (left). Histogram summarizes the effect of ELM exposure on spine density (right). (B) Bar histogram shows the effect of ELM exposure on the thin, mushroom and stubby spine density in apical dendrites. (C) Representative images of branched spines (shown in a red frame) on the 20 µm second-order dendrites of apical dendrites of control and ELM exposure group at 14 and 28 days respectively (left). ELM did not influence the branched spine density (right). *P<0.05; ^#^ *P*<0.05.

The majority of pyramidal neurons have a prominent thick apical dendrite which branches at, or is superficial to the border of layer I. The dendritic spines on the apical dendrite primarily form synapses with the axonal terminals from perirhinal and parahippocampal cortices [26]. In analyzing the spine in the apical dendrites, we found a significant reduction in spine density only at 28 days post ELM exposure (87.09±0.69%, *P*<0.05, Fig. 3A), while the changes observed at 14 days were not statistically different (101.82±3.41% of control, *P* = 0.65, Fig. 3A). We did not observe a significant change in the density of mushroom spines at 14 days after ELM exposure (115.59±1.99% of control, *P* = 0.23, Fig. 3B) and at 28 days after ELM exposure (88.64±0.70% of control, *P* = 0.21, Fig. 3B). Moreover, there was no significant change in the density of thin spines at 14 days (108.58±1.94% of control, *P* = 0.48; Fig. 3B) and in the density of stubby spines at 28 days (93.73±0.68% of control, *P* = 0.53; Fig. 3B). However, we found a significant reduction in stubby spines at 14 days (78.26±1.86% of control, *P*<0.05; Fig. 3B) and in thin spines at 28 days (74.37±0.59% of control, *P*<0.05; Fig. 3B). The density of branched spines was not affected by ELM at 14 days (*P* = 0.98, Fig. 3C) and 28 days (*P* = 0.27, Fig. 3C) after exposure.

**Figure 4:**
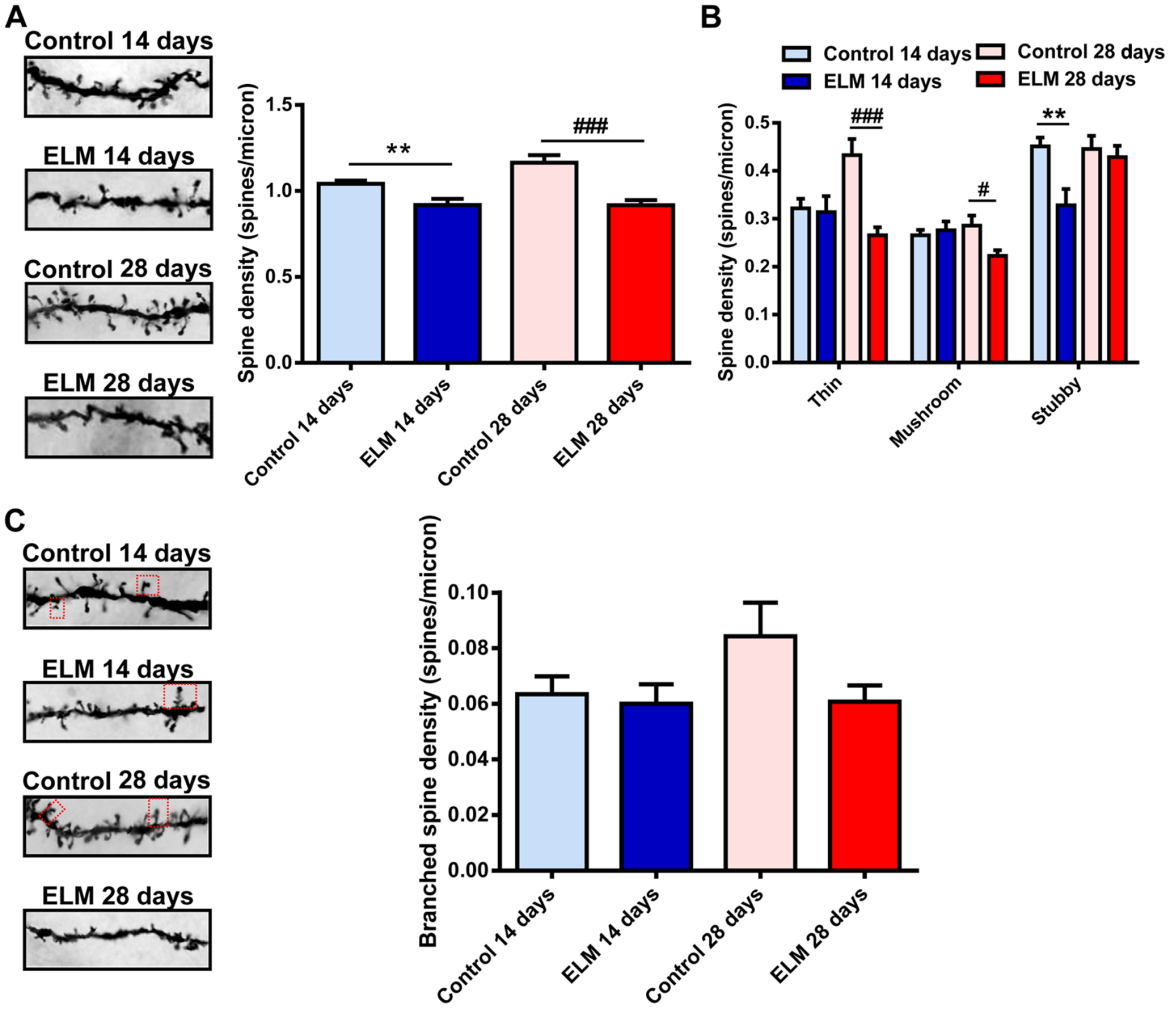
ELM exposure alters the dendritic spine density and shape in the basal dendrites of pyramidal neurons. (A) Representative images of spines on the 20 µm second-order basal dendrites (left). The histogram summarizes the effect of ELM exposure on spine density (right). (B) Bar histogram illustrates the effect of ELM exposure on the thin, mushroom and stubby spine density in basal dendrites. (C) Representative images of branched spines (shown in a red frame) on the 20 µm second-order dendrites of basal dendrites of control and ELM exposure group at 14 and 28 days respectively (left). ELM did not influence the branched spine density (right). **P<0.01; ^#^ *P*<0.05; ^###^ *P*<0.001.

The basal dendrites of pyramidal types were thin and short (Fig. 1C and Fig. 4A, 4C). They branch extensively within the layer III and mainly receive the inputs from the deep layers of the MEC [26]. Unlike the spines in the apical dendrites, spines in the basal dendrites were more sensitive to the ELM exposure. In the basal dendrites, there was a significant reduction in spine density at both 14 days (88.13±1.91% of control, *P*<0.01, Fig. 4A) and 28 days (78.73±0.67% of control, *P*<0.001, Fig. 4A) post ELM exposure. 14 days after ELM exposure, there was a significant reduction in the density of stubby spines (72.71±1.88% of control, *P*<0.01, Fig. 4B), while at 28 days the difference was not statistically significant (96.12±0.87% of control, *P* = 0.64, Fig. 4B). ELM exposure significantly decreased the density of thin and mushroom spines at 28 days (thin: 61.38±0.50% of control, *P*<0.001; mushroom: 77.88±0.56%, *P*<0.05; Fig. 4B). However, no significant difference was observed in the density of thin and mushroom spines at 14 days (thin: 97.55±1.66% of control, *P* = 0.85; mushroom: 103.88±1.64%, *P* = 0.66; Fig. 4B). Similar to the apical dendrites of pyramidal neurons, the density of branched spines was not affected by ELM at 14 days (*P* = 0.72, Fig. 4C) and 28 days (*P* = 0.09, Fig. 4C) after exposure.

## Discussion

In the present study, we showed that cranial ELM exposure altered the spine density and shape in the dendrites of stellate neurons and apical and basal dendrites of pyramidal neurons in the superficial layers of the MEC. There was a decrease in the spine density in the dendrites of stellate neurons and the basal dendrites of pyramidal neurons at both 14 days and 28 days post ELM exposure, which was predominantly attributed to the reduction of the thin spines. The decrease in the spine density in the apical dendrites of pyramidal neurons was only observed at 28 days after ELM exposure, suggesting that the different vulnerability between basal and apical dendrites. To our knowledge, our results for the first time reveal that ELM exposure affects the dendritic spine density and shape in the MEC. Considering the changes in spine shape and number correlate with synaptic plasticity and memory formation [20], [28], this may in part explain why ELM exposure impaired spatial learning and memory.

Almost all the fast synaptic activity in the cortex is mediated by the glutamatergic excitatory and GABAergic inhibitory synapses, and the MEC is no exception [11]. The extra-MEC excitatory inputs and local excitatory transmission in the MEC play a crucial role in the acquisition, consolidation and retrieval of spatial memory. The principal neurons in the superficial layers of the MEC receive visuospatial information from the neocortex primarily though excitatory synapses and then transform the information to all the subregions of hippocampus [13], [30], [31]. Furthermore, recent studies suggest that the formation of the spatial related-functional cells such as gird cells and grid × head direction conjunctive cells depends on the extra-MEC and local excitatory synaptic transmission [32], [33]. Given that the dendritic spines are the primary recipients of excitatory inputs and play an important role in determining neuronal input-output transformations, the pathological changes in spine number and shape post ELM exposure may disrupt the neural circuitry of the MEC and alter the firing properties of functional neurons, which may provide a neural mechanism at synaptic level for the ELM exposure-induced cognitive impairment.

One notable feature of dendritic spine is that they exhibit different morphologies. The variable spine shape and volume are thought to be correlated with different functional properties. It has been reported that the length of the spine neck affects calcium influx into the dendrite and thus might influence the activity mediated calcium transients in spines [34], [35]. Spines with large heads receive input from large axonal terminals containing more vesicles and exhibit higher glutamate sensitivity than the spines with small heads [36]–[38]. Moreover, In Schaffer collateral commissural synapses, the ratio of a-amino-3-hydroxy-5-methyl-4-isoxa-zolep-propionate (AMPA) to N-methyl-D-aspartic acid (NMDA) receptors is in proportion to the diameter of the post synaptic density (PSD) [39]. Considering the three types of spines classified by morphology may involve in different functional properties, each type of spine was analyzed separately in dendrites of stellate neurons and the apical and basal dendrites of pyramidal neurons.

The present study showed that the density of thin and branched spines was particularly decreased post ELM exposure in dendrites of stellate neurons and the basal dendrites of pyramidal neurons, reflecting a selective vulnerability of these spines to ELM exposure. Thin spines have been regarded as the ‘learning spines’ since they are highly dynamic and plastic and the alteration in their shape has been linked to different levels of recent synaptic activity [21], [40], [41]. Long-lasting enhancement of synaptic transmission may enlarge the head size of the thin spines and convert them to stable mushroom and branched spines [40]–[42]. Conversely, long-term depression of synaptic activity may shrink the dendritic spines and transform the mushroom and branched spines into thin spines [43], [44]. On the basis of observation that the decrease in the density of thin and branched spines, it is attempting to speculate that ELM exposure reduces the neuronal capacity for the structural plasticity in the MEC.

Unlike the effect on thin and branched spines, Long-term ELM exposure altered the density of mushroom and stubby spines in a cell-type specific manner. For the stellate neurons, ELM exposure slightly increased the density of stubby spines, while it did not affect the density of mushroom spines at 28 days post ELM exposure. It is conceivable that the change in the stubby spine density is a result of shrinkage of the thin spines. In the basal dendrites of pyramidal neurons, we observed a significant decrease in the mushroom spine density only at 28 days post ELM exposure. Additionally, the stubby spine density was reduced at 14 days and partially restored at 28 days post ELM exposure reflecting a compensatory mechanism. Mushroom spines have larger PSDs and contain more AMPA receptors [36], [45]. Compared to the thin spines, they are more likely to contain smooth endoplasmic reticulum for regulating the concentration of calcium and polyribosomes for local protein synthesis [46], [47]. Mushroom spines mediate stronger synaptic transmission and are thought to be ‘memory spines’ [21]. Thus, the reduction of mushroom spine density may reflect a detrimental effect of ELM exposure on the memory traces that have been already formed.

In the present study, we found the spines in the apical dendrites of pyramidal neurons were less sensitive to the ELM exposure than spines in the basal dendrites. Several lines of evidence suggest that the spines in the apical and basal dendrites exhibit distinct vulnerability. Chakraborti et al reported that cranial irradiation in young adult mice led to a decrease in spine density selectively in basal dendrites but not apical dendrites of CA1 pyramidal neurons [23]. Additionally, normal aging also caused a decrease in the spine density in basal but not apical dendrites in C57BL/6 mice [48]. Presently the mechanism underlying the distinct vulnerability in spine density between basal and apical dendrites remains unknown and additional studies are needed.
